# Assessment of Zearalenone-Induced Cell Survival and of Global Gene Regulation in Mouse TM4 Sertoli Cells

**DOI:** 10.3390/toxins14020098

**Published:** 2022-01-26

**Authors:** Christian Savard, Sadaf Gawhary, Alexandre Boyer, Younes Chorfi

**Affiliations:** Département de Biomédecine Vétérinaire, Faculté de Médecine Vétérinaire, Université de Montréal, 3200 rue Sicotte, Saint-Hyacinthe, QC J2S 7C6, Canada; christian.savard@biovet-inc.com (C.S.); sadaf.gawhary@umontreal.ca (S.G.); alexandre.boyer.1@umontreal.ca (A.B.)

**Keywords:** ZEA, mycotoxin, male reproduction dysfunction, TM4 cell line, sertoli cells

## Abstract

Zearalenone (ZEA) is a non-steroidal xenoestrogen mycotoxin produced by many *Fusarium* fungal species, which are common contaminants of cereal crops destined for worldwide human and animal consumption. ZEA has been reported in various male reproduction dysfonctions, including decreased fertility potential. In this report, the direct effect of ZEA on the immature Sertoli TM4 cell line was evaluated. The results show that high concentrations of ZEA increase reactive oxygen species via the activation of MAPK signaling. Transcriptome analysis was performed on the TM4 cell line treated with ZEA, and genes involved in sex differentiation (*Fgfr2*, *Igf1*, *Notch1*, *Sox9*) and extracellular matrix (ECM) formation (*Ctgf*, *Fam20a*, *Fbn1*, *Mmp9*, *Postn*, *Sparcl1*, *Spp1*) were identified at the center of the functional protein association network, suggesting that ZEA could be detrimental to the early steps of Sertoli cell differentiation.

## 1. Introduction

Sertoli cells are involved in testis biology and in the spermatogenesis process. During the testis development stage, Sertoli cells initiate the sex-specificity differentiation and coordinate the process of sex determination [1]. The subsequent proliferation of immature Sertoli cells until puberty determines sperm production capacity through adulthood, as each Sertoli cell is only able to sustain a limited number of germ cells, as the essential role of each Sertoli cell is to regulate germ cell number [2]. In the same way, mature Sertoli cells form an immunoprotective environment offer structural and nutritional support to germ cells [3].

Estradiol (E2) and its three receptors (ESR1, ESR2 and G protein-coupled estrogen receptor 1-GPER1) play a crucial role in the differentiation and maturation processes of Sertoli cells and also in the maintenance of homeostasis. It was proposed that E2 might interact with ESR1 in immature Sertoli cells to activate cell proliferation and with ESR2 to induce differentiation. Considering the pleiotropic role of E2, the ESR1/2 ratio is important to determine the dominating effect [4,5]. The importance of neonatal estrogen-mediated ESR1 signaling for spermatogenesis was also confirmed using a nonresponsive ESR1 knock-in mouse model [6], while GPER1 has been shown to regulate apoptosis in Sertoli cells [7]. These experiments suggest that estrogenic activity must be tightly regulated in the testis and in the Sertoli cells. That disturbing process due to endogenous estrogen or to xenoestrogens will affect Sertoli cell development and functions.

Zearalenone (ZEA) is a non-steroidal xenoestrogen mycotoxin produced by many *Fusarium* fungal species, which are common soil fungi and are prevalent in cereal growing regions. Due to intercropping and global food security procedures, humans and animals around the world may be affected [8]. Previous reports mention that the ZEA acts as an agonist on both ESRs and GPER1 and as an endocrine disruptor [9,10,11,12]. For these reasons, ZEA might affect the male reproductive system by causing testicular germ cell deficiency [13,14], reduced serum testosterone concentration [15] and reduced fertility [16]. The effect of ZEA on Leydig cells is of interest in research on the fertility of breeding animals. ZEA has been shown to directly inhibit androgen production induced by hCG or protein kinase A, to induce androgen production and to down-regulate the expression of several steroidogenic genes [15,17,18,19]. Fewer studies have evaluated its effects on Sertoli cells. It was demonstrated that the post-pubertal mice treated with ZEA from the embryonic stage led to a down-regulation of the expression of determining-region Y-box 9 (*Sox9*) and of Wilms tumor 1 (*Wt1*), two genes involved in the differentiation of Sertoli cells. These mutations on Sox9 and Wt1 genes might have occurred without, however, affecting the number of Sertoli cells in adult animals [20], while another study demonstrated that ZEA modulates the expression of the ABC transporters, proteins involved in the transport of nutrients, through the blood–testis barrier, both in vivo and in vitro [16]. Recent reports have mostly focused on identifying the mechanisms of action of ZEA on apoptosis and autophagy processes in Sertoli cells [17,21,22,23] using the TM4 Sertoli cell line as an in vitro model for proliferating immature Sertoli cells [24]. The objective of this study was, therefore, to obtain a more detailed transcriptomic analysis of ZEA action on the TM4 Sertoli cells.

## 2. Results

### 2.1. TM4 Immature Sertoli Cell Line Is Sensitive to ZEA

**Figure 1:**
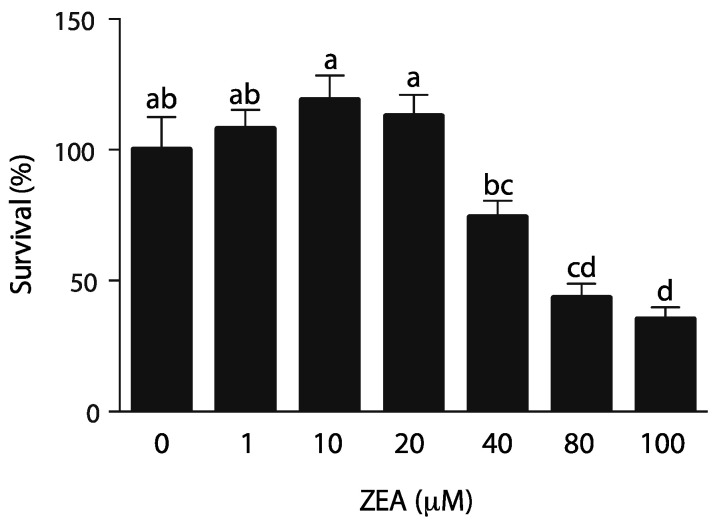
Effect of ZEA on TM4 cells viability. TM4 cells were incubated with increasing concentration of ZEA (0–100 µM) for 24 h. The concentration effect of the mycotoxin on cytoviability was quantified by assaying the quantity of purple-colored formazan product with an absorbance at 490 nm. Data are expressed as the means (±SEM) of survival (% of non-treated cells, exposure to 5% ethanol as vehicle). Values represent triplicates of three independent experiments. Data were analyzed by one-way ANOVA with Tukey’s multiple comparisons test. Letters a–d indicate significant difference between data sets (*p* < 0.05).

Proliferation of Sertoli cells during prepubertal testicular development is a key element to establish proper spermatogenesis, as each Sertoli cell can only support a defined number of germ cells. TM4 cells derived from Sertoli cells. To analyze the effect of ZEA on TM4 cell viability, cells from immature BALBc/mice were treated with increasing concentrations of ZEA (0. 1, 10, 20, 40, 80 and 100 µM) for 24 h. As shown in Figure 1, a slight increase, not significant, in cell viability was observed at 10 µM, while a significant decrease in cytoviability was observed at a concentration of 40 µM or higher and the IC_50_ of ZEA was estimated at 78 µM. The observed ZEA dose effect was consistent to the one previously observed by Long et al. [25] but differs from other groups in which cytoviability was affected at 10 µM [22] or at an even lower concentration affected starting at 1 µM [21].

### 2.2. Effects of ZEA on the MAPK Pathway

**Figure 2:**
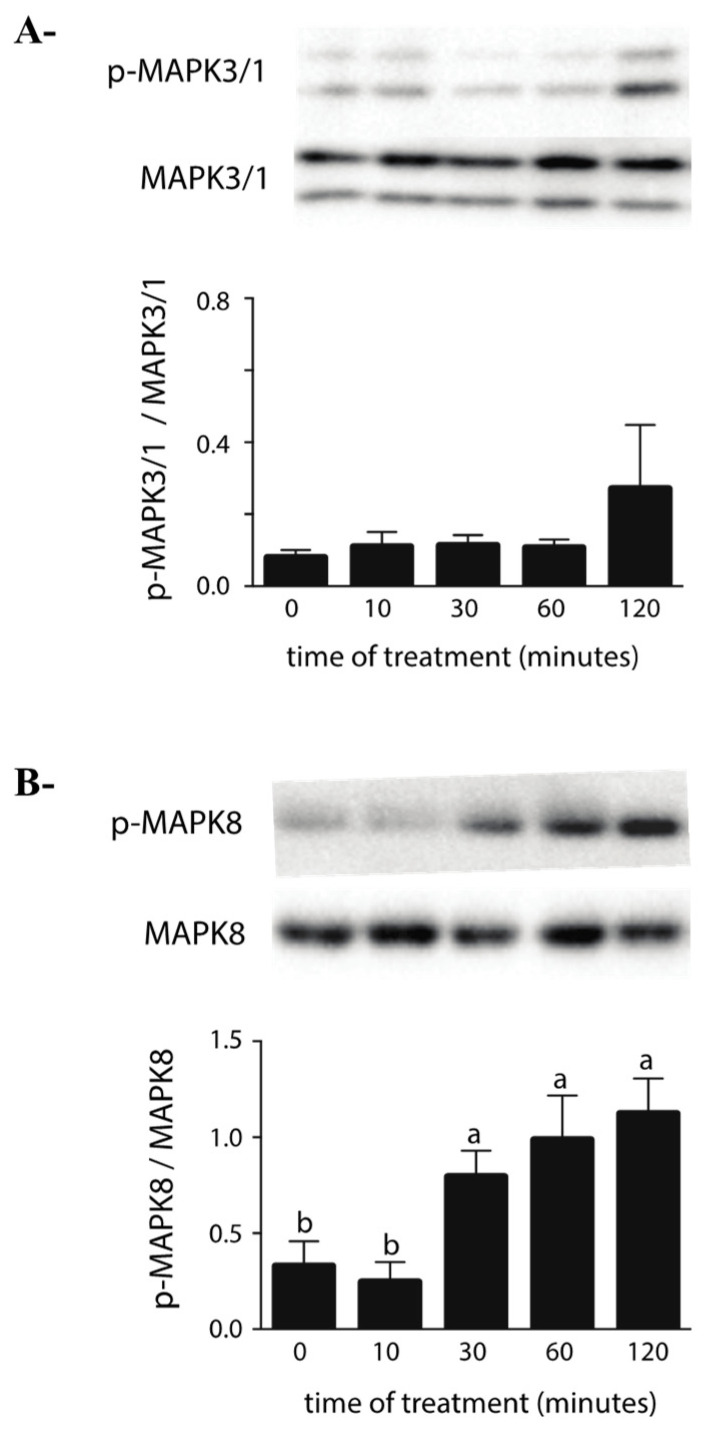

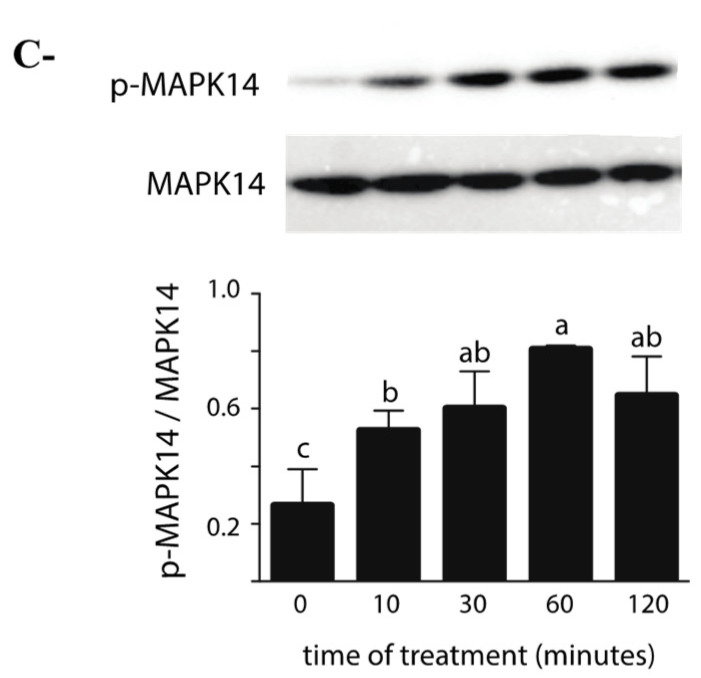
Effect of ZEA on MAPK signaling pathway. TM4 cells were treated with a fix concentration (80 µM) of ZEA up to 120 min. Western blotting with antibodies against total and phosphorylated forms of MAPK3/1 (**A**), MAPK14 (**B**) and MAPK8 (**C**) was performed. Representative blots from one replicate are shown above the corresponding diagrams. Data are represented as the ratio of phosphorylated total protein and are means (±SEM) of three independent cultures. Data were analyzed by one-way ANOVA with Tukey’s multiple comparisons test. Data labeled with superscripts of different letters indicate significant difference between data sets (*p* < 0.05).

Environmental toxicants have been shown to induce mitogen-activated protein kinases (MAPK) pathway activation and oxidative stress in the testis. ZEA has also been shown to induce apoptosis in some cell models via activation of MAPKs (MAPK8, MAPK14 and MAPK3/1) and oxidative stress-mediated signaling [26]. MAPK 8 is activated by stress and extracellular signaling and implicated in the apoptosis process, MAPK14 is activated by proinflammatory receptor stimulation and regulates autophagy via ROS formation and MAPK3/1 is activated by growth factor and inhibits cell proliferation and protein synthesis. Effects of high ZEA concentration on MAPK activation in the TM4 cell line were evaluated. Western blot analysis showed that exposure to 80 µM of ZEA induced a rapid increase in the p-MAPK8 to MAPK8 and p-MAPK14 to p-MAPK14 ratios, while the p-MAPK3/1 to MAPK3/1 ratio remained similar (Figure 2).

**Figure 3:**
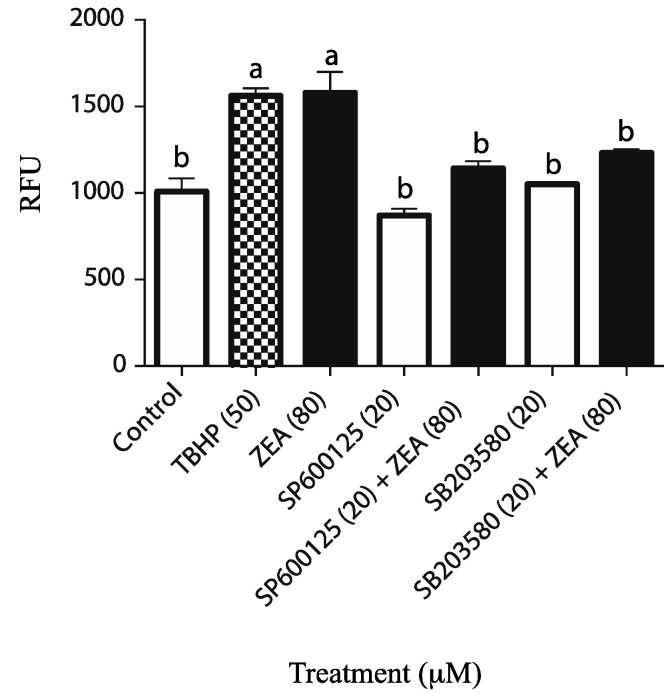
Effect of specific MAPK inhibitors on ROS production and gene expression. TM4 cells were previously incubated with 20 µM of specific SAP/JNK (SP600125) or P38 (SB203580) inhibitor for 1 h. Then, cells were treated with a fix concentration (80 µM) of ZEA for 1 h. The effect of selected inhibitors on ROS generated was assayed by a DCFDA-Cellular ROS detection assay. Tert-butyl hydrogenperoxide (TBHP) was used as a positive control. The values are expressed as the means (±SEM) of RFU and are obtained from triplicated of three independent experiments. Data were analyzed by one-way ANOVA with Tukey’s multiple comparisons test. Letters a to b indicate a significant difference between data sets (*p* < 0.05).

In order to verify if there was a correlation between ZEA-mediated MAPK activation and ROS generation, cells were exposed to 80 µM of ZEA in the presence or absence of MAPK inhibitors. ROS levels were quantified using the cell-permeant, 2′, 7′-dichlorofluorescein diacetate (DCFHDA) fluorescent probe. The results demonstrate that ZEA significantly increased ROS production (Figure 3), while ROS levels were significantly decreased in cells concomitantly treated with ZEA and MAPK8 (SP600125) or MAPK14 (SB203580) inhibitors (Figure 3), suggesting that MAPK signaling is involved in ROS generation by the potential induction of apoptosis by using a high concentration of ZEA.

### 2.3. Effects of ZEA on TM4 Cell Transcriptome

**Figure 4:**
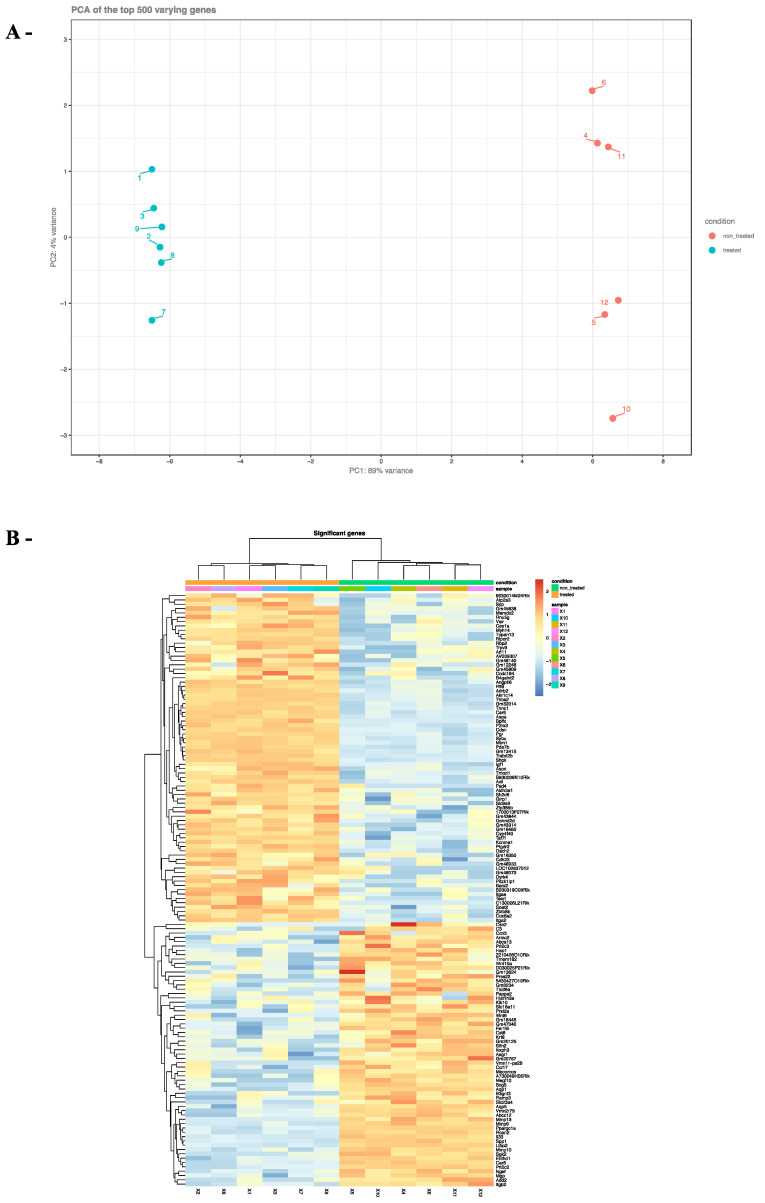
RNAseq analysis identified differentially expressed genes in TM4 cells exposed to ZEA. TM4 cells were exposed to ZEA (20 µM) for 24 h and RNAseq was performed on RNA extracted from six wells of ZEA-treated cells and six wells of vehicle (ethanol) generated from three different experiments. 2D-PCA biplot (**A**) and heatmap designed from the hierarchical clustering (**B**) illustrate two independent gene set populations. Variance in percentage is closed to the major component 1 and 2 (PC1, PC2). Selected genes for the heatmap have a log2 fold change higher than 4 and an adjusted *p*-value lower than 0.05.

To determine how ZEA affected the transcriptome of TM4 cells, cells treated with 20 µM of ZEA, the highest concentration that did not affect cell survival, (or vehicle) were analyzed by RNAseq. The quality of the RNAseq data was first evaluated using the main component analysis method. The 2D-PCA biplot generated two clear non-overlapping clusters corresponding to the TM4 cells treated with ZEA or vehicle, respectively, confirming that the variance between samples of the same group was small and that ZEA greatly affects the transcriptome of the TM4 cells (Figure 4A). These analyses differentiated 155 genes (least of 10 reads in either ZEA-treated or vehicle-treated samples) that were up-regulated by 2-fold or more in the ZEA-treated group and 134 genes that were down-regulated by 2-fold or more following ZEA treatment (Figure 4B, Table 1, Table 2 and Tables S1–S3—raw data).

**Figure 5:**
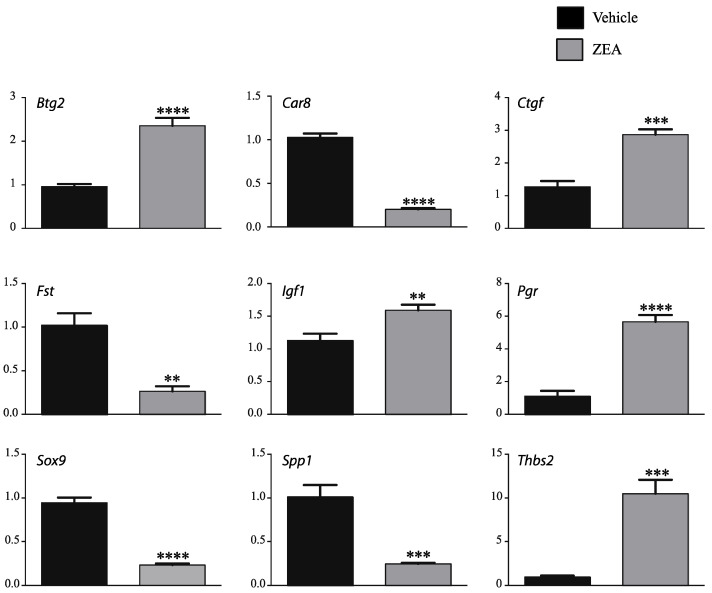
ZEA regulates expression of genes also regulated by estrogen signaling. Validation of the RNAseq data by RT-qPCR analysis (*n* = 5 ZEA-treated cells and *n* = 6 vehicle-treated cells). RT-qPCR data were normalized to the reference gene *Rpl19*. Data are expressed as mean (±SEM). Asterisks indicate significant differences from controls (** *p* < 0.01; *** *p* < 0.001; **** *p*< 0.0001).

To confirm the data set, mRNA levels of selected genes known to be regulated by E2 or to regulate *Esr1* [27,28,29,30,31,32,33,34,35,36,37,38,39] (B cell translocation gene 2, anti-proliferative (*Btg2*), carbonic anhydrase 8 (*Car8*), connective tissue growth factor (*Ctgf*), insulin growth factor (*Igf1*), progesterone receptor (*Pgr*), secreted phosphoprotein 1 (*Spp1*), SRY (sex-determining region Y)-box 9 (*Sox9*), thrombospondin 2 (*Thbs2*)) or important for Sertoli cell function or development [40,41,42,43,44,45] (Follistatin (*Fst*), *Igf1*, *Pgr*, *Sox9*) were verified by RT-qPCR (Figure 5). These results reveal the variations in gene expression that were comparable to the RNAseq findings except for *Igf1*, for which the fold change was reduced, but still statistically significant, by RT-qPCR (Table S5).

**Figure 6:**
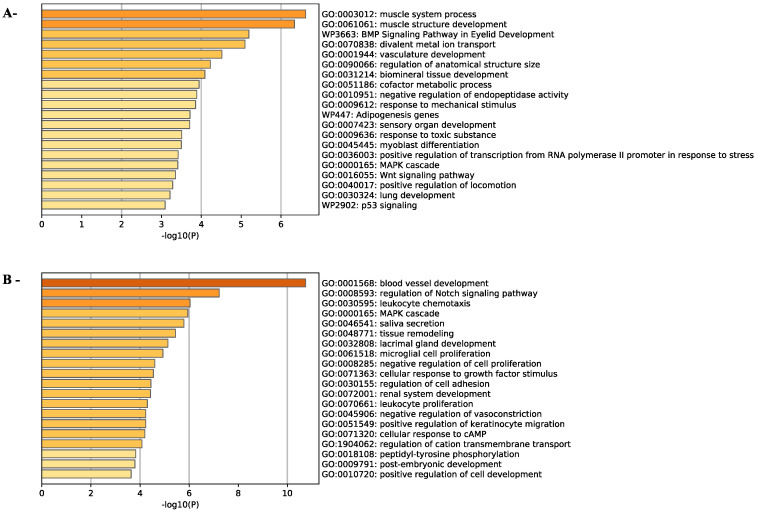
ZEA exposure mainly affects developmental gene expression in TM4 cells. Biological processes corresponding to the up-regulated (**A**) or to the down-regulated (**B**) genes in ZEA-treated TM4 cells using Metascape.

To study the biological processes involved by ZEA treatment, both up-regulated and down-regulated genes were subjected to gene ontology analysis using the Metascape gene annotation and analysis resources. This showed that ZEA mainly affected genes involved in development and cell proliferation and differentiation (Figure 6A,B). The expression of genes involved in response to a toxic substance and p53 signaling were also up-regulated in TM4 cells treated with 20 µM ZEA, suggesting that TM4 cells have initiated a toxicity response at this concentration (Figure 6A). Interestingly, genes in MAPK cascade involved in ROS formation are up-regulated, while the genes that inhibit ROS formation are down-regulated. (Figure 6A,B).

**Figure 7:**
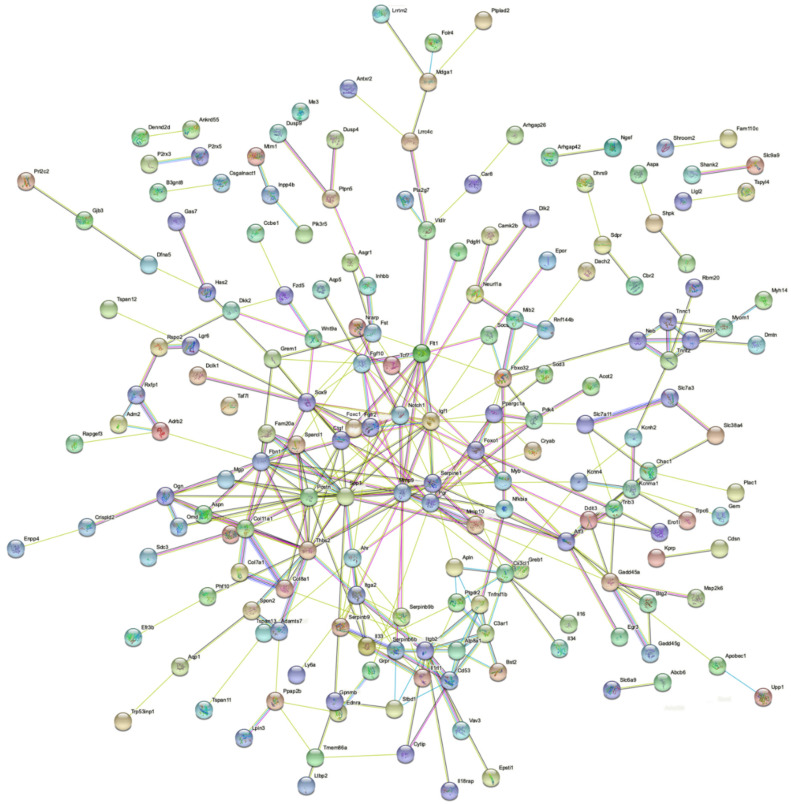
Gene network regulated by ZEA in TM4 cells. Gene network analysis of the up-regulated and down-regulated genes in ZEA-treated TM4 cells.

Finally, to understand the link between genes impacted by ZEA, a functional protein association network was generated with STRING resources. IGF1, NOTCH1, SPP1, MMP9, FGFR2, CTGF and SOX9 were among the genes identified at the center of the protein association network (Figure 7).

## 3. Discussion

ZEA is a non-steroidal xenoestrogen mycotoxin produced by *Fusarium* that has been shown to cause various disorders related to the reproductive system, including the Sertoli cell dysfunctions. In the present manuscript, the TM4 immature Sertoli cell line was used to further understand the hormetic concentration-dependent effect of ZEA in Sertoli cells.

To date, certain studies have evaluated the hormesis by ZEA on TM4 cells with divergent results concerning cell viability. The present data demonstrate a slight, but not significant, increase in cell viability at 10 µM followed by a significant decrease in cell viability at concentrations higher than 20 µM. These results are the same as those reported by Long et al. [25], who observed a rescue on cell viability at 10 µM but a marginal (>5%) statistically significant decrease in cell viability at 20 µM [46]. On the other hand, certain groups have shown a decrease in cell viability as early as 1 µM. These differences could be due to the presence of ethanol, used as vehicle to solubilize ZEA, the media culture, the source of ZEA and the method used to evaluate cell viability.

Previous studies have shown that ZEA can caused cell death by producing ROS and by inducing cell oxidative stress. In this report, we demonstrated that at high concentrations, ZEA might cause a rapid increase in the activation of both MAPK8 and MAPK14 signaling. Furthermore, MAPK8 and MAPK14 inhibitors are able to abolish the ROS production induced by ZEA. This data might reveal that MAPK signaling plays a key role in ZEA-induced oxidative stress. This is in accordance with previous studies that demonstrated that MAPK activation can induce apoptosis in numerous models (reviewed in [47]) and that MAPK activation by ZEA can also induce apoptosis [48] and ROS-related apoptosis in other cell models [26]. A previous study demonstrated that ZEA-induced ROS generation could also cause cell cycle arrest and apoptosis in the TM4 cells via AMPK signaling [22]. These results corroborate with the fact that MAPK8, 14 and AMPK have been shown to activate one another in different models [49,50,51,52,53].

In opposition to our study, it was demonstrated in a previous study that MAPK signaling was down-regulated in the TM4 cell line following ZEA exposure [21]. It is important to note that the concentrations of ZEA selected varied between both models (0.1 to 30 µM vs. 80 µM), as well as the time at which MAPK signaling was evaluated (24 h vs. 10 to 120 min), making it difficult to compare the resulting effects of these two models. Interestingly, genes associated with the signaling pathway involving MAPK are mainly down-regulated in our RNAseq data. The data acquisition was performed in the same conditions (20 µM, 24 h) to those used by Zheng et al. [21]. The differences in both models could be interpreted in the following two ways: first, that the activation of MAPK signaling by ZEA is robust but only transient in the TM4 cells; second, that ZEA regulates MAPK signaling differently depending on its concentration. Further experiments are essential to understand if the effect of ZEA on MAPK signaling is dose, time or both time and dose dependent. These experiments will be important as MAPK signaling and particularly MAPK8 and MAPK14 play important roles in controlling the balance between apoptosis and autophagy and can both positively and negatively promote autophagy depending on the context (reviewed in [54]).

Previous studies evaluating the effect of ZEA on TM4 immature Sertoli cells have mainly focused on its effects on autophagy and apoptosis. This study is the first to evaluate the global action of ZEA on these cells by RNAseq. Analysis of the RNAseq data demonstrated that genes, such as *Btg2* [27], genes regulated by estrogen in breast cancer protein (*Greb1*) [55,56], *Car8* [28,29], *Igf1* [30,31,32], *Pgr* [33,34], *Sox9* [35,36,37], *Spp1* [29] and *Thbs2* [38], to name a few, were regulated in a similar way by ZEA in TM4 cells and estradiol in varying systems, suggesting that ZEA acts at least in part via estrogen signaling. The functional association network identified two main categories of genes at the center of the network: genes associated with signalization (*Fgfr2*, *Igf1*, *Notch1*, *Sox9*) and genes associated with extracellular matrix (ECM) formation (*Ctgf*, Family with sequence similarity 20, member A (*Fam20a*), Fibrillin 1 (*Fbn1*), Matrix metallopeptidase 9 (*Mmp9*), Periostin (*Postn*), Sparc-like 1 (*Sparcl1*), *Spp1*). Inactivation of *Fgfr2, Sox9* and of the IGF1 receptors *Insr* and *Igf1r* in mouse models causes male-to-female sex reversal [43,44,57,58,59] and potentially also plays a role in the proliferation of pre-Sertoli/Sertoli cells [42,57,60], which is necessary for male fate of the gonads [61]. The constitutive activation of NOTCH1 in Sertoli cells causes gonocyte exit from quiescence and entry into meiosis, usually observed in the developing ovary [62,63]. Therefore, down-regulation of *Fgfr2* and *Sox9* and up-regulation of *Notch1* in ZEA-treated TM4 cells all suggest that ZEA affects the male identity of the cells, while an increase in *Igf1* could suggest that TM4 cells try to compensate for this loss. The role of individual ECM-related genes in immature Sertoli cells have not been clearly defined. However, it was demonstrated that ECM components were expressed in Sertoli/granulosa cells in a sex-specific pattern, with *Ctgf*, *Postn* and *Spp1* all being mainly expressed by developing Sertoli cells [64]. It was also demonstrated that external supply of ECM gels around pre-Sertoli cells can restore *Sox9* expression in gonads with reduced *Sox9* expression and enhanced testis-cord formation [65] and that components of the ECM also regulate the sex-specific activity of FGF9, the ligand of FGFR2, in Sertoli cells [60]. This suggests that proper ECM constitution is important for de novo tubulogenesis [66].

Based on the role of the genes at the center of the network, ZEA might be particularly detrimental for testis differentiation. This analysis is in accordance with a previous report, which demonstrated that exposure to xenoestrogen exacerbates male reproductive system disorders such as hypospadias, cryptorchidism, testicular germ cell cancer and low sperm counts and that developing testes are more sensitive to estrogens during the early fetal period [67,68]. Although, these problems have been mainly associated with the fetal Leydig cells implicated in the deficiency in testosterone production [68]. In addition, Sertoli cells are essential for androgen production both directly, as the conversion of androstenedione to testosterone occurs within the Sertoli cells in the fetal testes [69], and indirectly, as paracrine signals secreted by Sertoli cells are essential for fetal Leydig cell development [70,71].

In conclusion, this study shows pleiotropic effects of the mycotoxin ZEA in an immature Sertoli cell line and suggests that ZEA might interfere with Sertoli cell development.

## 4. Materials and Methods

### 4.1. Cells

TM4 mouse Sertoli cell line was purchased from ATCC and maintained for 24 h at 37 °C in a 5% CO_2_ incubator in DMEM/F12 medium (Life Technologies, Burlington, ON, Canada) supplemented with 100 U/mL penicillin, 100 µg/mL streptomycin, 0.25 µg/mL fungizone (Life Technologies), 15 mM HEPES, 5% horse serum and 2.5% FBS.

### 4.2. Determination of Viable Proliferating Cells

Cells at final concentration of 1 × 10^4^ were seeded in 96-well plates, then cells were treated with various concentrations (1, 10, 20, 40, 80 and 100 M) of ZEA or with vehicle and were incubated for 24 h (37 °C, 5% CO_2_). Cell viability was assayed with CellTiter 96^®^ Aqueous One Solution Cell Proliferation Assay (Promega, Madison, WI, USA). A total of 20 µL of the CellTiter 96^®^ was used. A fixed volume of substrate was added to the cells and incubated for 1 h at 37 °C in a humidified 5% CO_2_ incubator. Optical density of test and control wells was acquired at 490 nm using a Synergy™ HT multi-detection microplate reader (Biotek, Winooski, VT, USA). Experiments were performed three times in triplicates, and data are expressed as percentage of control cells.

### 4.3. Western Blot Analysis

After ZEA exposure, cells were washed with cold PBS and lysed in SDS loading buffer (50 mM Tris-HCl, pH6.8, 2% SDS, 10% glycerol, 1% β-mercaptoethanol, 12.5 mM ethylene diamine tetra-acetic acid and 0.02% bromophenol blue). Samples were separated via 10% SDS–PAGE and blotted onto Hybond-P PVDF membrane (GE Amersham, Piscataway, NJ, USA). After transfer, the membranes were blocked in TTBS-BSA (10 mM Tris–HCl, 150 mM NaCl, 0.1% Tween-20, 5% BSA, pH 7.5) for 1 h at room temperature. Membranes were incubated overnight at 4 °C with the primary antibody against mitogen-activated protein kinase 8, 14 and 3/1 (MAPK8, MAPK14, MAPK1/3) (# 9252, 9215, 9102) and their phosphorylated form (#9251, 9211, 9101) (Cell Signaling Technology, Danvers, MA, USA) diluted 1:1000 in TTBS-BSA. After three washing steps with TTBS, membranes were incubated for 1 h at 25 °C with 1:10,000 anti-rabbit HRP-conjugated IgG (W401B) (Promega, Madison, WI, USA) diluted in TTBS-5% non-fat dry milk. After three washes in TTBS, protein bands were visualized by chemiluminescence using Immobilon western chemiluminescent HRP substrate (Millipore, Etobicoke, ON, Canada) and quantified using a ChemiDoc MP detection system and Image Lab™ software (Bio-Rad, Mississauga, ON, Canada).

### 4.4. Detection of Cellular ROS

Cells were maintained in 96-well plates as described above and pre-treated for 1 h with 20 µM of inhibitors specific to MAPK8 (SP600125) or MAPK14 (SB203580) purchased from Selleckchem (Houston, TX, USA). Control cells were pre-treated with DMSO. Then, cells were washed and stained with 25 µM DCFDA (Abcam, Toronto, ON, Canada) for 45 min at 37 °C. The unbond dye was removed by an additional washing step, before the cell treatment with 80 µM of ZEA for 1 h or 50 µM of tert-butyl hydrogenperoxide (TBHP) for the positive control. After 1 h of incubation, relative fluorescent unit (RFU) was acquired directly in microplate, at Ex 485 nm/Em 535 nm, using a Synergy™ HT multi-detection microplate reader (Biotek, Winooski, VT, USA). Experiments were performed three times in triplicates.

### 4.5. RNAseq and RT-qPCR Analyses

TM4 cells 2.5 × 10^5^ were seeded in 6-well plates. After 24 h of incubation, cells were treated with ZEA (20 µM) or vehicle for 24 h. Total RNA was extracted using the RNeasy mini kit (Qiagen, Toronto, ON, Canada) according to the manufacturer’s protocol. Extracted RNA was resuspended in 30 µL of RNase free water and freezed until RNAseq or RT-qPCR analyses. RNA samples were transferred to the genomics core facility of the Institute for Research in Immunology and Cancer (IRIC, Montreal, QC, Canada) for RNAseq analyses. RNA quality and quantity were assayed with an Agilent 2100 Bioanalyzer using the RNA 6000 Pico kit (Agilent Technologies, Santa Clara, CA, USA). Twelve RNA-seq libraries (*n* = 6 ZEA treated cells and *n* = 6 vehicle treated cells) were generated using the KAPA mRNA Hyperprep (poly-A capture) libraries (Roche, Mississauga, ON, Canada). Single-read (1X 75 base pairs, maximum 1X 85 bp) sequencing was performed on a Nextseq500–0.5 Flowcell High Output (20M reads per sample) (Illumina, San Diego, CA, USA). Sequences were trimmed of sequencing adapters and low-quality 3’ bases using Trimmomatic version 0.35 [72] and aligned to the reference mouse genome version GRCm38 (gene annotation from Gencode version M23, based on Ensembl 98) using STAR version 2.7.1a [73]. Gene expression resulted from both a direct readcount from STAR as well as computed using RSEM [74] in order to obtain a normalized gene and a transcript level expression in TMP values for these non-stranded RNA libraries. DeSeq2 version 1.22.2 [75] was then used to normalize gene readcount. Data analysis from the Metascape gene annotation and analysis resource led to the evaluation of the biological processes regulated by the up- or down-regulated genes (2-fold or more) [76] or processed through STRING functional protein association network [77].

For validation of the RNAseq results, quantification of RNA was performed using a Nanodrop (NanoDrop Technologies, Inc., Wilmington, DE, USA). A total of 100 ng of total RNA was reverse-transcribed using the SuperScript^®^ VILO™ cDNA Synthesis Kit (Life Technologies, Burlington, ON, Canada) according to the manufacturer’s instructions. Real-time PCR reactions were run on a CFX96 Touch instrument (Bio-Rad, Hercules, CA, USA), using Supergreen Advanced qPCR MasterMix (Wisent, St-Bruno, QC, Canada). Each PCR reaction consisted of 7.5 μL of Power SYBR Green PCR Master Mix, 2.3 μL of water, 4 μL of cDNA sample, and 0.6 μL (400 nM) of gene-specific primers. PCR reactions run without complementary cDNA (water blank) were reported as negative controls. A thermal cycling program (3 min at 95 °C, 40 cycles of 15 s at 95 °C, 30 s at 60 °C and 30 s at 72 °C) was used to amplify each transcript. To quantify relative gene expression, the Ct of genes of interest was compared with that of ribosomal protein L19 (*Rpl19*), according to the ratio R = [E^Ct *Rpl19*^/E^Ct target^], where E is the amplification efficiency for each primer pair. Any significant variation was not observed about *Rpl19* Ct between tissues or cells, and *Rpl19* was, therefore, deemed suitable as an internal reference gene. The specific primer sequences used for RT-qPCR are mentioned in Table S1.

### 4.6. Statistical Analysis

All statistical analyses were performed using GraphPad Prism software (version 5.03, GraphPad Prism software Inc., San Diego, CA, USA). Data were statistically analyzed using a one-way ANOVA with Tukey’s multiple comparison test, except RT-qPCR data, which were analyzed using a Student’s *t*-test. All the data sets were subjected to the F test to determine the equality of variances prior to statistical testing. All data are presented as means ± standard deviation of the mean. Means were considered significantly different when *p* values were <0.05.

## Figures and Tables

**Table 1:** List of the top 35 up-regulated genes in TM4 cells treated with 20 μM of zearalenone. The genes confirmed by qPCR are represented in bold.

Gene	Fold-Change	Padj
tgdr2	13.22	1.10 × 10^−13^
Kcnma1	13.19	4.57 × 10^−13^
Avil	12.45	2.17 × 10^−14^
Car6	10.74	6.92 × 10^−35^
Slc9a9	10.18	1.55 × 10^−09^
Shpk	10.16	5.42 × 10^−148^
Myh14	9.19	3.55 × 10^−22^
Taf7l	8.40	1.56 × 10^−15^
**Thbs2**	**8.03**	**2.73 × 10^−72^**
Gm32014	7.98	3.04 × 10^−63^
Ripor2	7.78	2.20 × 10^−27^
Gm43314	7.47	5.40 × 10^−11^
Akr1c14	7.34	2.98 × 10^−49^
Tnnc1	7.14	4.15 × 10^−65^
Itga2	7.10	7.99 × 10^−20^
P2rx3	6.77	2.07 × 10^−49^
Pde7b	6.58	6.72 × 10^−32^
Cdsn	6.53	1.39 × 10^−112^
B930036N10Rik	6.22	2.32 × 10^−21^
Aspn	5.95	3.09 × 10^−10^
Aspa	5.93	1.16 × 10^−132^
Gm13415	5.75	8.28 × 10^−26^
Zbtb8b	5.60	2.23 × 10^−26^
**Igf1**	**5.58**	**1.90 × 10^−33^**
Trabd2b	5.57	4.33 × 10^−146^
Dach2	5.51	1.31 × 10^−16^
Adrb2	5.46	9.24 × 10^−58^
Tspan13	5.32	2.63 × 10^−08^
Glrp1	4.76	4.20 × 10^−06^
Angptl6	4.58	3.93 × 10^−21^
Dennd2d	4.51	1.19 × 10^−09^
Gm16485	4.44	1.029 × 10^−06^
Tmod1	4.39	2.60 × 10^−11^
Sybu	4.38	1.80 × 10^−65^
**Pgr**	**4.34**	**4.36 × 10^−75^**

**Table 2:** List of the top 35 down-regulated genes in TM4 cells treated with 20 µMof zearalenone.

Gene	Fold-Change	Padj
Aqp1	−26.28	9.45 × 10^−15^
Prl2c2	−19.87	1.87 × 10^−69^
Fer1l6	−12.35	6.90 × 10^−26^
Mmp10	−10.47	8.09 × 10^−13^
**Car8**	**−6.93**	**6.11 × 10^−32^**
Il33	−6.72	5.82 × 10^−168^
Ppargc1a	−6.08	9.72 × 10^−100^
Vmn2r79	−5.94	7.08 × 10^−23^
Scg5	−5.02	1.56 × 10^−14^
Aqp5	−4.86	7.48 × 10^−08^
Asgr1	−4.48	5.97 × 10^−05^
**Spp1**	**−4.43**	**1.24 × 10^−198^**
Ngef	−4.43	5.69 × 10^−12^
Ltbp2	−4.42	9.61 × 10^−125^
Itgb2	−4.38	4.24 × 10^−11^
Enthd1	−4.31	2.07 × 10^−07^
Gm47040	−4.30	3.48 × 10^−08^
Mgp	−4.15	1.37 × 10^−14^
Rcan2	−4.15	2.24 × 10^−66^
Mmp9	−4.12	2.75 × 10^−30^
Tcim	−3.94	3.24 × 10^−115^
Inpp4b	−3.87	5.52 × 10^−42^
**Fst**	**−3.77**	**3.50 × 10^−251^**
Tmem179	−3.77	3.31 × 10^−07^
Apln	−3.73	1.42 × 10^−174^
Ahr	−3.72	2.16 × 10^−15^
Tspan11	−3.60	7.52 × 10^−117^
Neto2	−3.57	9.91 × 10^−75^
**Sox9**	**−3.56**	**1.28 × 10^−26^**
Ccdc88c	−3.52	1.19 × 10^−12^
Junos	−3.50	5.81 × 10^−12^
Epsti1	−3.44	1.21 × 10^−07^
Tmem200a	−3.43	7.27 × 10^−11^
Gas1	−3.42	9.01 × 10^−93^

## Supplementary Materials

The following supporting information can be downloaded at: https://www.mdpi.com/article/10.3390/toxins14020098/s1, Table S1: List of the up-regulated genes in TM4 cells treated with 20 µM of zearalenone (2-fold or more, minimum 10 reads), Table S2: List of the down-regulated genes in TM4 cells treated with 20 µM of zearalenone (2-fold or more, minimum 10 reads), Table S3: Raw data, Table S4: Comparison of fold-change in mRNA levels (ZEA treated vs. non-treated cell) for selected genes, as determined by RNAseq and RT-qPCR, Table S5: Quantitative RT-PCR primer sequences.
