# Supplementary material for: Assessment of Zearalenone-Induced Cell Survival and of Global Gene Regulation in Mouse TM4 Sertoli Cells
Source: Toxins (Basel). 2022 Jan 26;14(2):98. doi: 10.3390/toxins14020098 (PMC8874968; doi:10.3390/toxins14020098)
Supplement: Supplementary file 1 [file toxins-14-00098-s001.zip › Table S4 and S5.pdf]

Supplementary Materials: Assessment of Zearalenone-Induced Cell Survival and of Global Gene Regulation in Mouse TM4 Sertoli Cells

Christian Savard, Sadaf Gawhary, Alexandre Boyer and Younes Chorfi

Table S4. Comparison of fold-change in mRNA levels (ZEA treated vs non-treated cell) for selected genes, as determined by RNAseq and RT-qPCR.

Gene	RT-qPCR fold-change	RNAseq fold-change	Average reads in ZEA treated cells	Average reads in control cells
Btg2	2,45	2,23	446.6 ± 13.8	199.4 ± 7.3
Car8	-5,08	-6,93	9.4 ± 1.1	65.29 ± 3.4
Ctgf	2,25	2,03	26508 ± 538.3	13050 ± 497.5
Fst	-3,87	-3,77	462.6 ± 12.21	1745 ± 50.9
Igf1	1,48	5,58	149.8 ± 26.5	14.51 ± 3.9
Pgr	5,14	4,34	216.9 ± 3.9	49.53 ± 3.8
Sox9	-4,11	-3.56	29.1 ± 3.1	103 ± 6.7
Spp1	-4,15	-4,43	1738 ± 53.0	7691 ± 395.4
Thbs2	11,22	8,03	44913 ± 1986	5505 ± 584.6

Table S5. Quantitative RT-PCR primer sequences.

Gene	Forward	Reverse
Btg2	GTGGGTTTCCTCTCCAGTCTC	CCAGTGGTGTTTGTAATGATCGGT
Car8	GATGTCCGCCTGTCTCCAAA	TGGTCCTCCTGACAAGACTGA
Ctgf	GAGGAAAACATTAAGAAGGGCAAAA	CCGCAGAACTTAGCCCTGTA
Fst	CAAAGTCCTGTGAAGATATCCAGT	TAGGAAAGTTGTAGTCCTGGTCT
Igf1	AAGGCAGTTTACCCAGGCTC	TCTTTATTGCAGGTGCGGTCA
Pgr	TCCAGGTGACCCATGAGGAA	TTGCCTTGATCAATTCGCGG
Rpl19	CTGAAGGTCAAAGGGAATGTG	GGACAGAGTCTTGATGATCTC
Sox9	TGCTGCTTCGACATCCACACGTG	GACGTGCAAGCTGGCAAAGTTGA
Spp1	CCTTGCTTGGGTTTGCAGTC	TGGTCGTAGTTAGTCCCTCAGA
Thbs2	TGGCATCGCTGTAGGTTTCG	TGCCACTGAAGTCCACAGAC
